# Pseudomonas aeruginosa C-Terminal Processing Protease CtpA Assembles into a Hexameric Structure That Requires Activation by a Spiral-Shaped Lipoprotein-Binding Partner

**DOI:** 10.1128/mbio.03680-21

**Published:** 2022-01-18

**Authors:** Hao-Chi Hsu, Michelle Wang, Amanda Kovach, Andrew J. Darwin, Huilin Li

**Affiliations:** a Department of Structural Biology, Van Andel Institute, Grand Rapids, Michigan, USA; b Department of Microbiology, New York University Grossman School of Medicine, New York, New York, USA; Weill Cornell Medical College

**Keywords:** C-terminal processing protease, cell wall, cryo-EM, Pseudomonas aeruginosa, structural biology

## Abstract

Pseudomonas aeruginosa CtpA is a carboxyl-terminal processing protease that partners with the outer membrane lipoprotein LbcA to degrade at least five cell wall-associated proteins, four of which are cell wall hydrolases. This activity plays an important role in supporting P. aeruginosa virulence in a mouse model of acute pneumonia. However, almost nothing is known about the molecular mechanisms underlying CtpA and LbcA function. Here, we used structural analysis to show that CtpA alone assembles into an inactive hexamer comprising a trimer of dimers, which limits its substrate access and prevents nonspecific degradation. The adaptor protein LbcA is a right-handed open spiral with 11 tetratricopeptide repeats, which might wrap around a substrate to deliver it to CtpA for degradation. By structure-guided mutagenesis and functional assays, we also showed that the interfaces of the CtpA trimer of dimers and an N-terminal helix of LbcA are important for LbcA-mediated substrate degradation by CtpA both *in vitro* and *in vivo*. This work improves our understanding of the molecular mechanism of the LbcA-CtpA proteolytic system and reveals some striking differences from the arrangements found in some other bacterial CTPs.

## INTRODUCTION

A eubacterial cell is protected by a mesh-like cell wall of peptidoglycan (PG), which is composed of linear glycan strands with peptide side chains that cross-link with peptide bonds (1). To accommodate growth, these cross-links must be cleaved so that nascent PG can be inserted into the network (1–3). Several PG endopeptidases carry out this hydrolysis, including MepS, MepM, and MepH in Escherichia coli (4, 5). However, if not tightly regulated, their activity could lead to rupture of the PG sacculus and cell death. One way to control endopeptidase activity is through the relatively recently discovered carboxyl-terminal processing (CTP) protease system. CTPs belong to the S41 family of serine proteases (6). All CTPs have a PDZ domain—so named because it was first noted in postsynaptic density protein of 95 kDa, *Drosophila* disc large tumor suppressor, and zonula occludens-1 protein—which plays roles in substrate recognition and protease regulation (7, 8). CTPs work within the cell envelope of Gram-negative and Gram-positive bacteria and have been linked to virulence (9–14).

**FIG 1:**
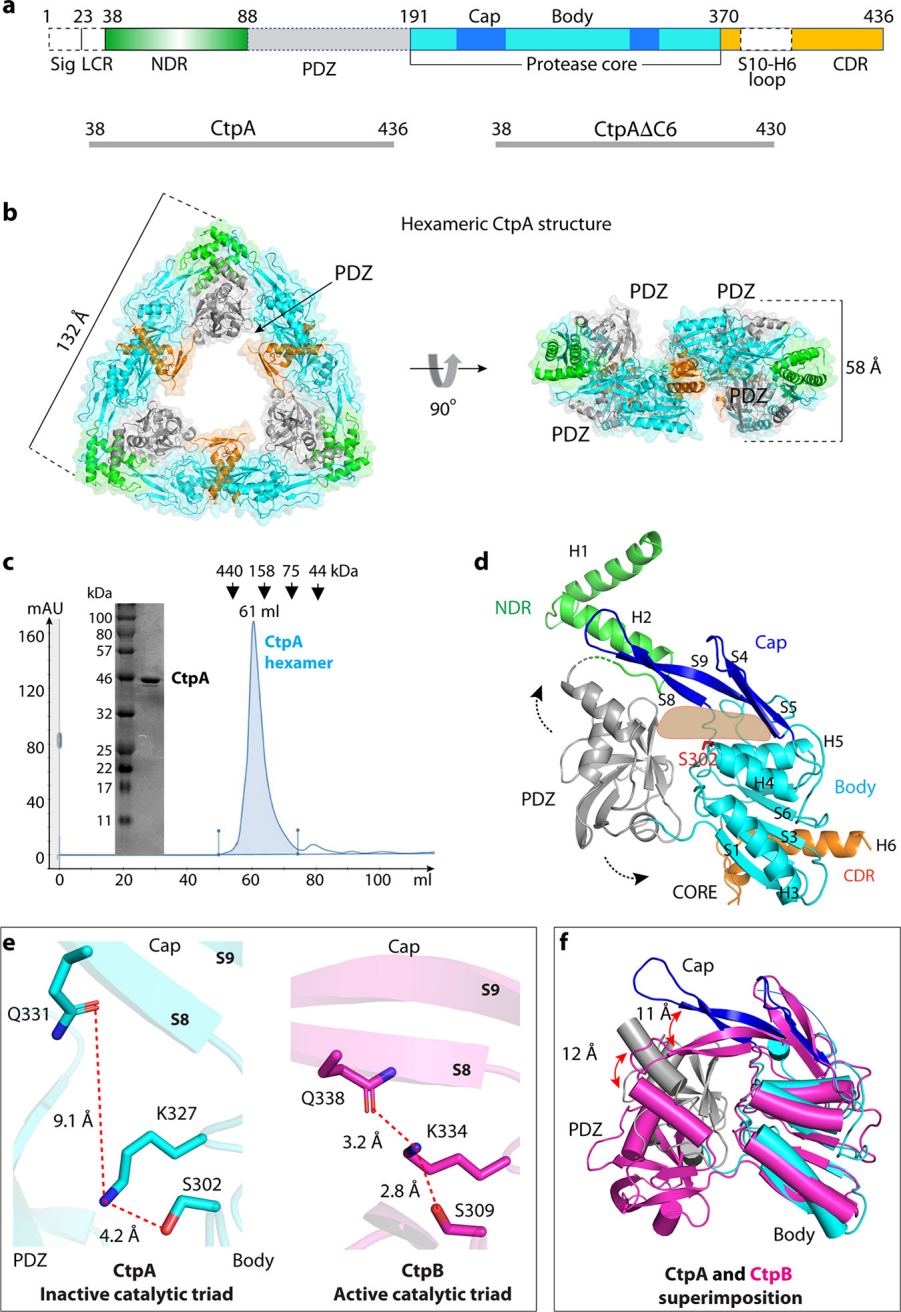
Overall structure of CtpA. (a) Top: CtpA domain organization. The dashed lines indicate the disordered regions (PDZ and the S10-H6 connecting loop) that are not resolved in the crystal structure. NDR = N-terminal dimerization region; CDR = C-terminal dimerization region; Sig = signal sequence; LCR = low complexity region. The sequence ranges of the two CtpA constructs used this study are shown in the lower panels. (b) Cartoon and transparent surface views of CtpA hexamer. The domains are colored according to the depiction in panel a. (c) SDS-PAGE analysis and gel filtration profile of CtpA. mAU = milli-absorbance units; kDa = kiloDaltons. (d) A CtpA subunit in cartoon view. Secondary structural elements in CtpA are labeled, except in the PDZ domain. The two dashed arrows indicate the mobile PDZ domain in the CtpA hexamer. The light orange shape indicates the tunnel between the cap and the body region. (e) Comparison of the catalytic triads of P. aeruginosa CtpA and B. subtilis CtpB (PDB ID 4C2E). The S8 and S9 labels refer to β-strands 8 and 9 in the cap region. (f) Superposition of the core domains of inactive P. aeruginosa CtpA (cyan) and active B. subtilis CtpB (magenta). Catalytic Ser-302 in CtpA and Ser-309 in CtpB are in red sticks. Red arrows indicate the lifted-up (CtpA) and clamped-down positions (CtpB) of the PDZ and the cap subdomains.

The E. coli CTP Prc partners with the lipoprotein Nlpl to cleave the PG endopeptidase MepS (5). Prc is a bowl-shaped monomer, and the Nlpl adaptor forms a homodimer that binds to two separate molecules of Prc (15). In Bacillus subtilis, the CtpB protease processes and activates the intramembrane protease 4FA-4FB complex, thereby regulating spore formation (16, 17). CtpB has N-terminal and C-terminal dimerization domains, plus a cap domain preceding the protease core domain. Structural analysis revealed that CtpB assembles a dimeric self-compartmentalizing ring structure (18). The substrate peptide enters the proteolytic site via a narrow tunnel that is largely sequestered by the PDZ domain and becomes exposed only in the presence of a substrate. Therefore, the CtpB protease is reversibly activated by the substrate C-terminal peptide. In contrast to Prc and CtpB, CtpA of Pseudomonas species has been assigned to the C-terminal processing peptidase-3 group (6, 19) (Fig. 1A). No structural studies have been reported so far for this group.

Pseudomonas aeruginosa is an opportunistic human pathogen. It is one of the leading causes of sepsis in intensive care units, and outbreaks of multidrug-resistant strains have been reported in hospitals (20–22). In contrast to E. coli, in which the only CTP present is Prc, P. aeruginosa has both the C-terminal processing peptidase-1 group member Prc and the C-terminal processing peptidase-3 group member CtpA (10, 19). We reported previously that CtpA is required for normal function of the type 3 secretion system and for virulence in a mouse model of acute pneumonia (10). P. aeruginosa CtpA has 39% amino acid sequence identity with B. subtilis CtpB, which suggests that they might share a similar fold. However, CtpA has a longer C-terminal region. This extended C terminus might alter the oligomerization mode relative to that of CtpB (18). However, it is not known whether or how CtpA assembles into a self-compartmentalizing structure to prevent nonspecific proteolysis. Unlike CtpB, CtpA is not directly activated by a protein substrate. Instead, it requires the adaptor protein LbcA, the lipoprotein-binding partner of CtpA, for activity *in vivo* and *in vitro* (23).

LbcA is a predicted outer membrane lipoprotein containing 11 tetratricopeptide repeats (TPRs). The TPR motif is a degenerate 34-amino acid sequence that mediates protein-protein interactions (24–26). LbcA promotes CtpA protease activity, and *ctpA* and *lbcA* null mutants share common phenotypes, such as a defective type 3 secretion system and accelerated surface attachment (10, 23, 27). Five LbcA-CtpA substrates have been reported to date, and four of them are predicted to be PG cross-link hydrolases: the LytM/M23 family peptidases MepM and PA4404 and the NlpC/P60 family peptidases PA1198 and PA1199 (23, 27). Therefore, it appears that LbcA interacts with CtpA to assemble an active proteolytic complex, which controls the activity of these enzymes by degrading them. However, the molecular mechanisms underlying CtpA and LbcA function are unknown. Here, we describe structural and functional analyses of CtpA and LbcA. We show that CtpA alone assembles as an inactive trimer of dimers and that LbcA is a right-handed spiral that might wrap around a substrate protein. Structure-guided mutagenesis confirms the functional importance of CtpA interfaces and identifies the interface between CtpA and LbcA.

## RESULTS

### CtpA assembles a trimer-of-dimers hexamer in solution.

CtpA is located in the periplasm, tethered to the outer membrane via its interaction with LbcA (23). Its N-terminal 23 residues are a type I signal sequence, followed by a 14-residue region that is enriched in alanine, glycine, and proline that contributes to a bacterial low complexity region and is predicted to be disordered (28). Therefore, we removed these 37 amino acids to produce a CtpA protein for structural and functional studies (Table S1; Fig. 1a). For simplicity, we refer to this ΔN37 construct as CtpA throughout the main text. The CtpA crystals diffracted X-rays to 3.5-Å resolution. We used molecular replacement with the B. subtilis CtpB as a search model to solve the CtpA structure, but this did not lead to a satisfactory solution. We then produced selenomethionine-substituted CtpA crystals for single-wavelength anomalous diffraction (SAD)-based phasing. The derivatized crystals diffracted to 3.3 Å, leading to successful structural solution (Table S2). There are two CtpA molecules in one asymmetric unit (ASU), and CtpA formed a hexamer that comprised a trimer of dimers in crystal (Fig. 1b). This oligomer state is consistent with an estimated mass of a hexamer from the gel filtration profile (Fig. 1c). As expected, each CtpA protomer consists of an N-terminal dimerization region (NDR), a PDZ domain, a cap domain, a protease core domain, and a C-terminal dimerization region (CDR) (Fig. 1d). The PDZ domain is partially disordered, but we generated a homolog PDZ model based on the CtpB structure and accurately docked the domain guided by two anomalous density peaks from selenomethionine residues 153 and 160 of the domain (Fig. S1). The loop connecting S10 and H6 (amino acids [aa] 378–411) within the CDR was disordered, and the last two residues (435 and 436) were not resolved.

10.1128/mbio.03680-21.1 TABLE S1 Strains and plasmids used in this study Table S1, PDF file, 0.06 MB. Copyright © 2022 Hsu et al. 2022 Hsu et al. https://creativecommons.org/licenses/by/4.0/ This content is distributed under the terms of the Creative Commons Attribution 4.0 International license.

10.1128/mbio.03680-21.2 TABLE S2 Crystallographic data collection and refinement statistics Table S2, PDF file, 0.04 MB. Copyright © 2022 Hsu et al. 2022 Hsu et al. https://creativecommons.org/licenses/by/4.0/ This content is distributed under the terms of the Creative Commons Attribution 4.0 International license.

10.1128/mbio.03680-21.3 FIG S1 Se-Met peaks superimposed on the PDZ homolog model. **a**) The Se-Met peaks are superimposed on the CtpA atomic model shown in ribbons. The Se positions are shown as orange spheres. The two CtpA subunits are in in yellow and green. The two PDZ domains are in grey. **b**) The electron density map of one asymmetric unit obtained from the Se-derivatized CtpA crystal. The 2mFo−DFc electron density map is rendered at 1σ threshold and shown as blue meshes. The positions of Se are in orange. **c**) The SAD-map obtained from Autosol in Phenix. The two low occupancy Se positions in PDZ are show as orange spheres. The docked Met153 and Met160 of PDZ are shown in gray. Download FIG S1, JPG file, 0.5 MB. Copyright © 2022 Hsu et al. 2022 Hsu et al. https://creativecommons.org/licenses/by/4.0/ This content is distributed under the terms of the Creative Commons Attribution 4.0 International license.

### The CtpA hexamer alone in the absence of LbcA is in an inactive configuration.

The CtpA core region contains the protease-active site. There is a narrow tunnel dividing the core domain into the upper cap and lower body regions (Fig. 1d). In CtpB, this tunnel was suggested to guide the substrate peptide into the proteolytic site (18). The cap of CtpA is a four-stranded β-sheet (S4, S5, S8, and S9) located below the NDR. The body region is composed of a three-helix bundle (H3, H4, and H5) and a five-stranded β-sheet (S1, S2, S3, S6, and S7). The CtpA catalytic Ser-302 is located at the end of the narrow tunnel between the cap and the body region.

The CtpA hexamer is in an inactive conformation. The PDZ domain is in a position that blocks the narrow substrate peptide tunnel leading to Ser-302. Furthermore, the catalytic triad Ser-302–Lys-327–Gln-331 is well beyond hydrogen-bonding distance: Ser-302 and Lys-327 are 4.2 Å apart, and Lys-327 and Gln-331 are 9.1 Å apart (Fig. 1e). We solved the crystal structure of CtpA that was catalytically inactive due to an S302A substitution and found that it was also in the inactive configuration (Fig. S2). For comparison, the catalytic triad Ser-309–Lys-334–Gln-338 in the protease-active B. subtilis CtpB are all within hydrogen-bonding distance (Fig. 1e). By superimposing the two proteases, we found that transition to the active form requires the CtpA cap domain to clamp down toward the catalytic site by 11 Å and also a large-scale movement of the associated PDZ domain by 12 Å (Fig. 1f; Video S1). The CtpA structure is consistent with our previous observation that purified CtpA alone was inactive in degrading its substrates (23). The addition of purified LbcA was able to activate the protease of wild-type CtpA but not of the mutant CtpA(S302A). Therefore, unlike CtpB, which fluctuates between an active and inactive form in solution and can be activated by a protein substrate (18), the CtpA hexamer is locked in an inactive configuration and requires LbcA for activation (23).

10.1128/mbio.03680-21.4 FIG S2 Crystal structure of CtpA(S302A) showing only a monomer. (A) overall structure of CtpA(S302A) in ribbons, with the catalytic triad residues in sticks and the point mutation S302A in red sticks. (B) Enlarged view of the catalytic triad in the CtpA(S302A) crystal structure. The structure is also in the inactive configuration. Download FIG S2, JPG file, 0.1 MB. Copyright © 2022 Hsu et al. 2022 Hsu et al. https://creativecommons.org/licenses/by/4.0/ This content is distributed under the terms of the Creative Commons Attribution 4.0 International license.

10.1128/mbio.03680-21.5 VIDEO S1 CtpA hexamer structure. Rotation of the hexamer, then transition to a monomer to show the domain structure, then transition to morphing between inactive and active computational model (based on CtpB). Download VIDEO S1, MOV file, 6.4 MB. Copyright © 2022 Hsu et al. 2022 Hsu et al. https://creativecommons.org/licenses/by/4.0/ This content is distributed under the terms of the Creative Commons Attribution 4.0 International license.

### The C-terminal dimerization interface is important for full CtpA activity.

**FIG 2:**
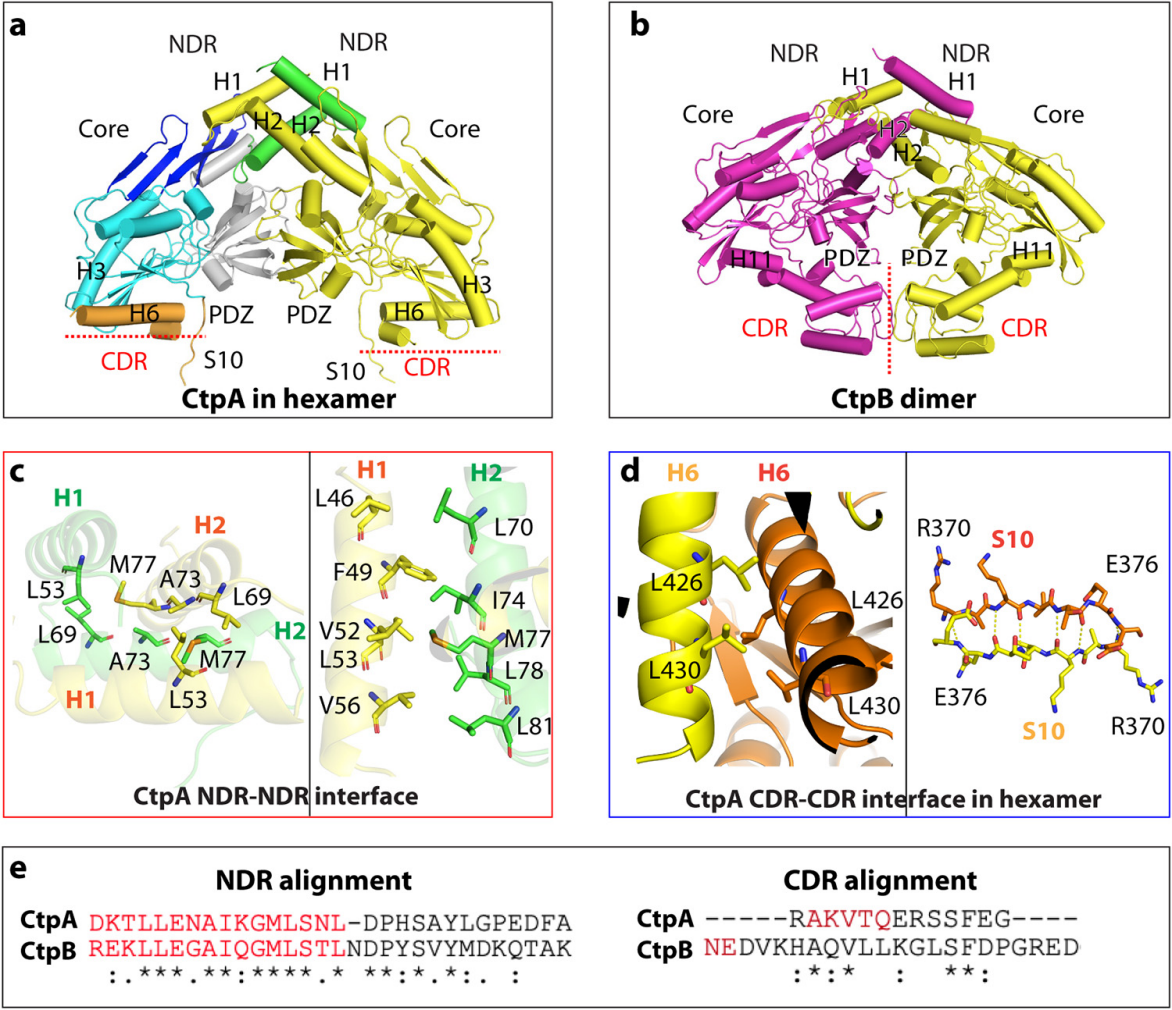
Different oligomerization modes of P. aeruginosa CtpA and B. subtilis CtpB. (a) CtpA dimer extracted from the CtpA hexamer. (b) B. subtilis CtpB dimer (PDB ID 4C2E). CtpB H11 is the equivalent of CtpA H6. (c) The N-terminal dimerization interface of CtpA involves hydrophobic interactions between two H2s (left) and between H1 and H2 (right). (d) The C-terminal dimerization interface of CtpA involves a short leucine zipper-like interaction between two H6 helices and antiparallel β-sheet formation between two S10s. (e) Alignment of the conserved NDR sequence and divergent CDR between P. aeruginosa CtpA and B. subtilis CtpB.

The N terminus of P. aeruginosa CtpA dimerizes via its NDR in the same way as B. subtilis CtpB (Fig. 2a). However, the six unhinged CDRs of three CtpA dimers interact to form the triangular trimer-of-dimer complex (Fig. 1b and 2a). In contrast, CtpB forms a parallel homodimer by both head-to-head N-terminal and tail-to-tail C-terminal interactions (Fig. 2b) (18). The CtpA NDR is composed of the H1 and H2 helices. The NDRs of two CtpA monomers form a domain-swapped, intermolecular four-helix bundle, in which the NDR of one protomer reaches over to contact the cap domain of the other protomer (Fig. 2a and c). The four-helix bundle is at the vertex of the triangular complex. The NDR-NDR dimerization is driven by hydrophobic interactions. Specifically, Leu-69, Ala-73, and Met-77 are at the intersecting point of the two crossed H2s. Met-77 hydrophobically interacts with Ala-73 and Leu-69 of the second protomer (Fig. 2c). H1 of protomer 1 is nearly parallel to H2 of protomer 2, with the H1 residues Leu-46, Phe-49, Val-52, Leu-53, and Val-56 hydrophobically interacting with the H2 residues Leu-70, Ile-74, Met-77, Leu-78, and Leu-81. The Leu-46 and Phe-49 of the two H1s are also within the van der Waals distance. The hydrophobic NDR-NDR interaction of the CtpA dimer resembles that in the CtpB dimer, consistent with the conserved sequence in this region (Fig. 2e).

**FIG 3:**
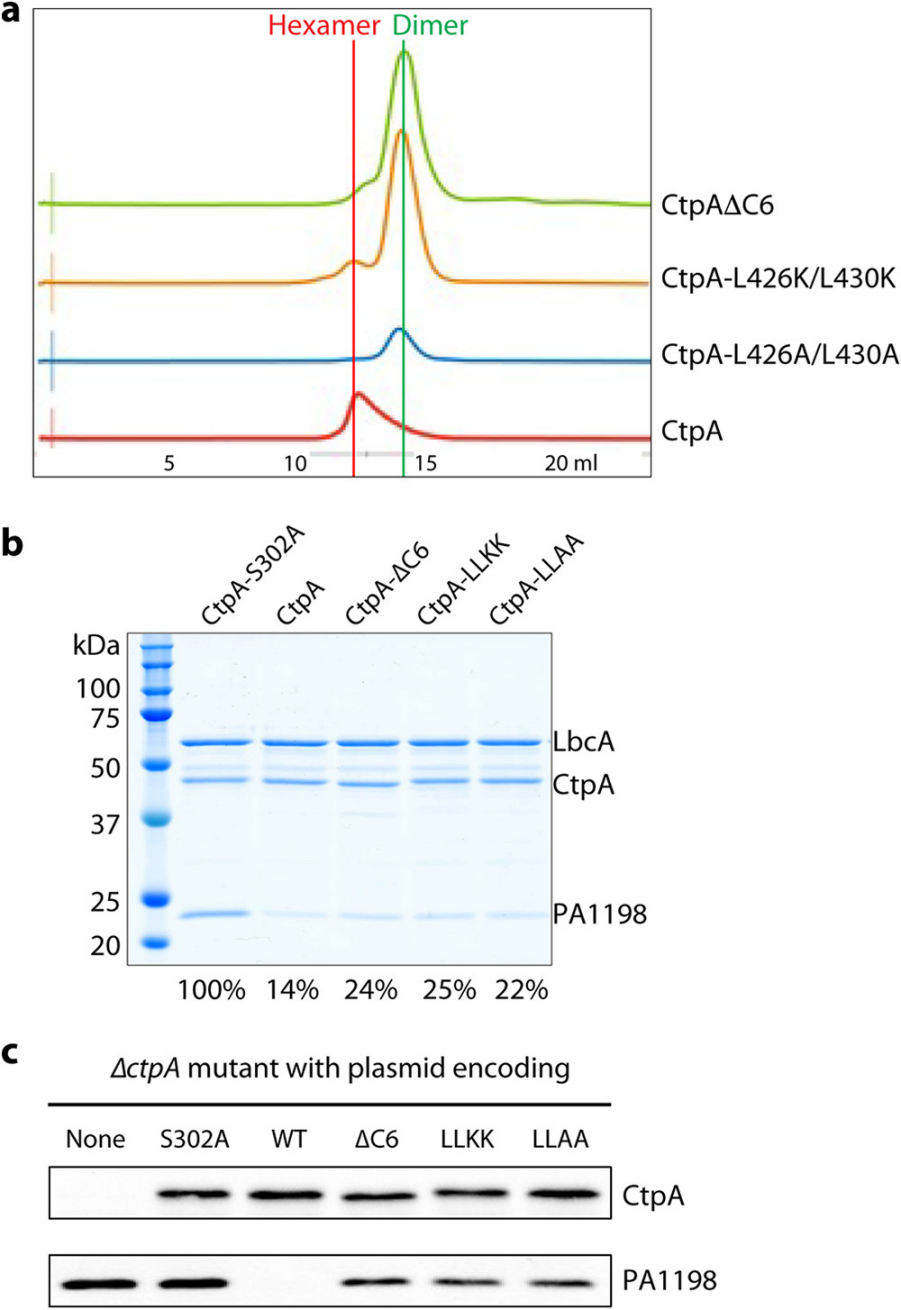
Full protease activity of CtpA requires C-terminal dimerization region. (a) Elution profiles of wild-type and mutant CtpA proteins. (b) Substrate degradation assay *in vitro*. His_6_-PA1198 served as the substrate for the assay. Gels from a single experiment are shown, but the amount of PA1198 degradation is the average from two independent experiments, determined as described in the Materials and Methods section. The number below each lane is the percentage of remaining PA1198 after 3 h, relative to the first lane using the inactive CtpA. (c) Substrate degradation *in vivo*. Plasmid-encoded protease-dead CtpA(S302A), wild type (WT), ΔC6, L426K/L430K (LLKK), and L426A/L430A (LLAA) were produced in a P. aeruginosa Δ*ctpA* strain. None = empty plasmid vector control. The CtpA proteins and accumulation of the PA1198 substrate were detected by immunoblot analysis with polyclonal antisera.

The C-terminal dimerization interface of CtpA involves the β-strand S10 and the helix H6 (Fig. 2d). The two S10 β-strands (Arg-370 to Glu-376) form an intermolecular, antiparallel β-sheet that reinforces the dimer interface. The two H6s (Tyr-418 to Gly-435) are orthogonal to each other but form a short leucine zipper in the middle section mediated by Leu-426 and Leu-430. The CtpA C-terminal dimer interface is not like that of CtpB, which dimerizes via interaction in a different region. Indeed, the C-terminal sequence of P. aeruginosa CtpA is not conserved in B. subtilis CtpB (Fig. 2e), which is consistent with their different oligomeric assembly. To investigate the functional importance of the unique C-terminal dimerization interface of CtpA, we constructed a mutant with a partially disrupted H6 helix by removing the last six residues Ser-431 to Asn-436; CtpA(ΔC6). We found that CtpA(ΔC6) eluted from a gel filtration column as a dimer (Fig. 3a), so the C-terminal truncation prevented CtpA hexamer formation.

We next asked whether the C-terminal dimerization interface, present in the intact CtpA hexamer but disrupted in the CtpA(ΔC6) dimer, is required for normal CtpA function. In an *in vitro* assay, we found that the CtpA(ΔC6) dimer was less active than the CtpA hexamer in degrading the model substrate PA1198, in reactions that also contained LbcA (compare lanes 3 and 4 in Fig. 3b).

To further investigate the functional significance of the leucine zipper in H6, we generated two CtpA double mutant constructs with L426K/L430K and L426A/L430A substitutions that disrupted the leucine zipper interaction. The two purified mutant proteins were mainly dimeric and failed to assemble into a hexamer, based on their gel filtration profiles (Fig. 3a). Their LbcA-activated *in vitro* protease activity toward PA1198 was lower relative to wild-type CtpA (Fig. 3b, compare lanes 5 and 6 with lane 3). However, like the CtpA(ΔC6) mutant, the leucine zipper mutants retained significant protease activity relative to the protease dead mutant CtpA(S302A) (Fig. 3b, compare lanes 5 and 6 with lane 2).

We extended our analysis by constructing plasmids encoding derivatives of full-length CtpA with the ΔC6, L426K/L430K (LLKK), or L426A/L430A (LLAA) mutations. After introducing these plasmids into a P. aeruginosa Δ*ctpA* mutant, immunoblot analysis showed that the steady-state levels of the mutant CtpA proteins were similar to the wild type (Fig. 3c). However, PA1198 accumulated in the presence of these three mutants relative to the wild type, although not as much as in the presence of CtpA(S302A) (Fig. 3c). This suggests that the protease activity of the three mutants was reduced, but not abolished, which is consistent with the *in vitro* analysis (Fig. 3b). Therefore, our studies suggest that the hexameric assembly is important for proper CtpA function.

### LbcA forms a spiral that could wrap around CtpA substrates.

**FIG 4:**
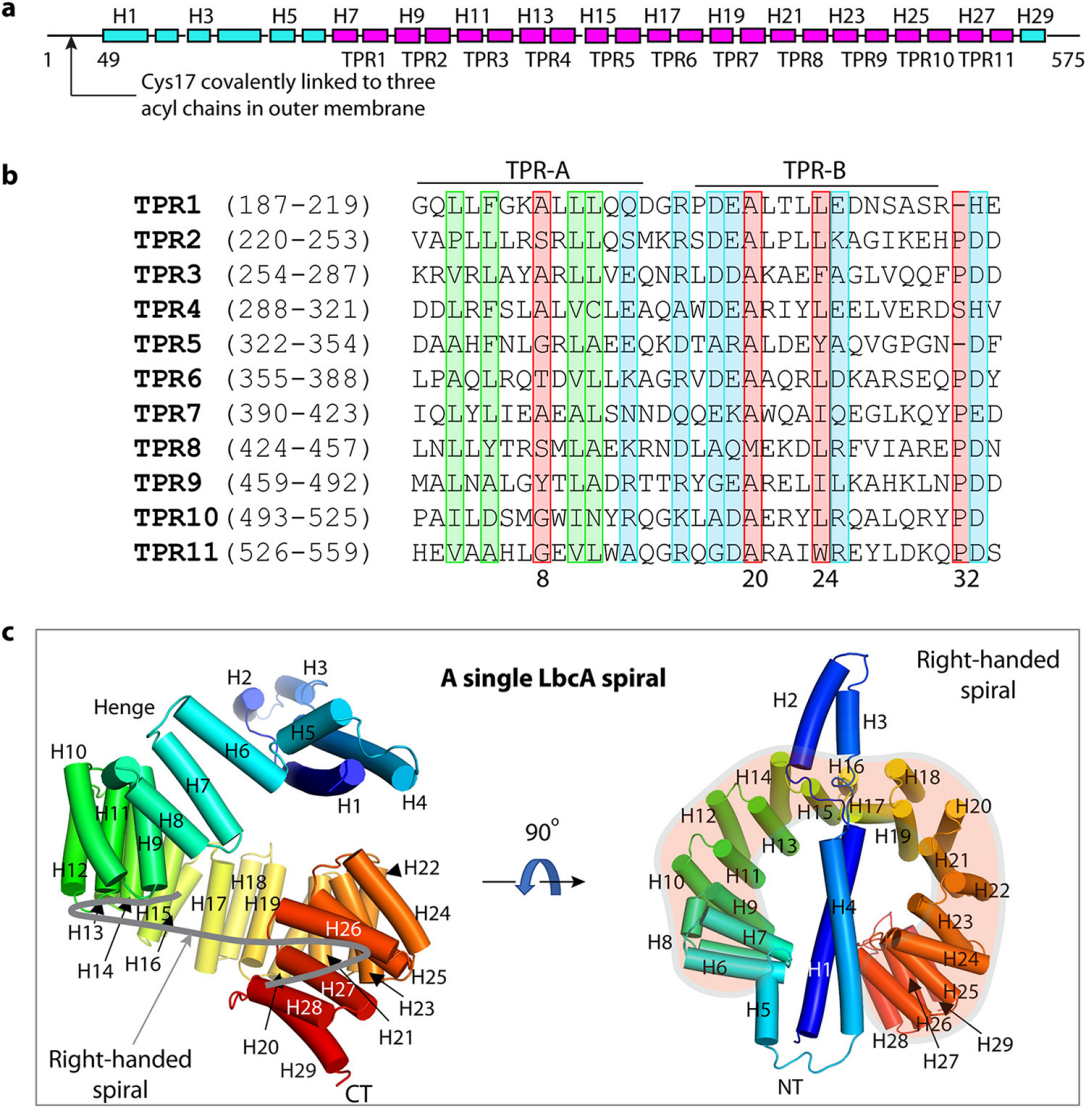
Overall structure of LbcA. (a) Domain organization of LbcA. The TPR motifs are shown in magenta. TPR = tetratricopeptide repeats. (b) Sequence alignment of the 11 TPRs of LbcA. The signature residues of TPR are marked in the red rectangles. The highly homologous hydrophobic residues are in green boxes, and the highly homologous charged residues are in the blue boxes. (c) The crystal structure of LbcA(ΔN48) contains an N-terminal extension with 4 helices and a C-terminal superhelix composed of 11 TPRs. The thick gray curve in the left panel follows the right-handed spiral feature of the TPRs.

During the maturation of the lipoprotein LbcA, its N-terminal 16 residues are removed, exposing Cys-17 (Fig. 4a). Then Cys-17 is lipidated so that the N terminus can be anchored in the outer membrane from the periplasmic side (29–31). LbcA is able to bind to CtpA and its substrates independently (27). Therefore, to begin to understand how LbcA might be capable of these separate interactions, we tried to solve the LbcA crystal structure. We were able to crystallize LbcA with its N-terminal 48 residues removed (ΔN48). The crystal structure was solved to 3.5-Å resolution by the SAD method with selenomethionine-derivatized LbcA(ΔN48) crystals (Fig. 4a to c). This structure contains only α-helices and connecting loops, with a total of 29 α-helices. We resolved all 11 predicted tetratricopeptide repeats, comprising 22 α-helices from helix 7 (H7) to H28. The sequences of the 34-residue TPRs are largely conserved (Fig. 4b). Gly/Ala at position 8 and Ala at positions 20 and 27 are most conserved in the TPR family, although none of these are invariant. Interestingly, TPR1, TPR5, and TPR10 contain only 33 amino acids, but they all contain the signature residues Gly/Ala at position 8 and Ala at position 20, and TPR1 and TPR10 also contain a signature Leu at position 24. Within each TPR, the first helix (TPR-A) lines the inner surface, and the second helix (TPR-B) lines the outer surface of the ring structure. Alignment of the 11 TPRs in LbcA(ΔN48) showed that more-conserved hydrophobic residues are concentrated in the TPR-A helix and more-conserved charged residues are distributed in TPR-B helix and in the turns connecting TPR-A and TPR-B, suggesting that the inner and the outer surfaces of the ring may have distinct functions.

The 11 TPRs form a notched ring, with an outer diameter of about 6 nm and an inner diameter of about 3 nm (Fig. 4c). It is a right-handed spiral structure, because the first TPR is slightly above the ring, and the last TPR is below the ring. The first four α-helices at the N terminus form an elongated extension that partially caps the TPR ring. Helices H5 and H6 serve as a hinge that links the N-terminal helical extension and the TPR spiral. However, the loop connecting H4 and H5 (aa 163 to 171) has a high crystallographic B-factor (150 to 200 Å^2^) that is indicative of flexibility. Therefore, this loop and the H5-H6 hinge are likely to allow relative motion of the NT helical extension with respect to the TPR spiral. The TPR ring is capped at the end by the single short α-helix, H29.

**FIG 5:**
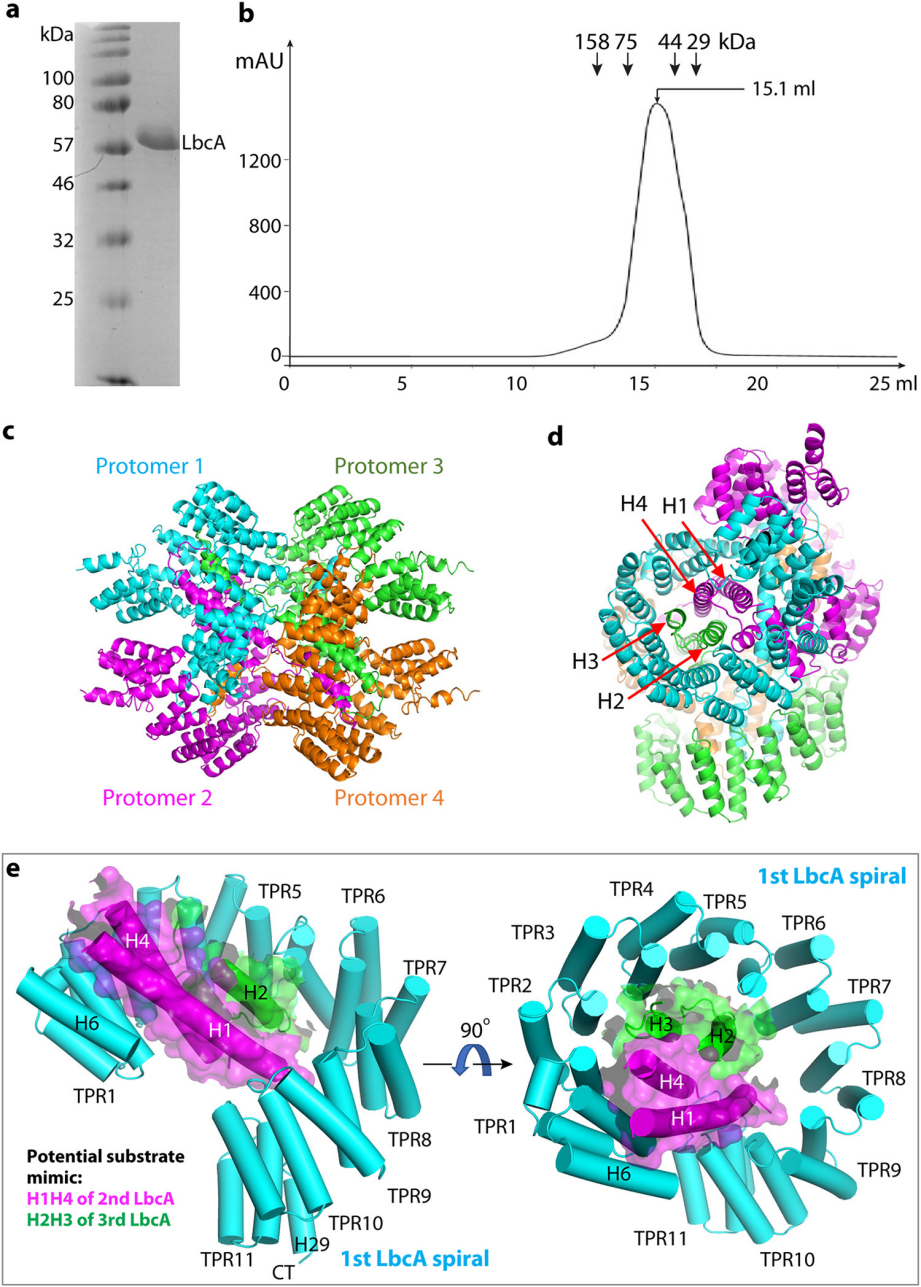
LbcA is a monomer in solution but assembles a tetramer in crystal. (a) Coomassie blue-stained SDS-PAGE gel of the purified of LbcAΔN48. (b) Superdex 200 elution profile of LbcAΔN48. LbcA was eluted from a gel filtration column at a volume corresponding to the monomeric state. (c) LbcA forms an intertwined tetramer in the crystal lattice. The four protomers are individually colored. (d) This LbcA tetramer view shows that the helices H1 and H4 of protomer 2 and helices H2 and H3 of protomer 3 form a 4-helix bundle inside the super helical coil of protomer 1 in the crystal lattice. (e) H1H4 of a second LbcA (magenta cartoon and transparent surface) and H2H3 of a third LbcA (green cartoon and transparent surface) insert into the first LbcA spiral, likely mimicking the substrate binding by the first LbcA spiral.

LbcA(ΔN48) purified as a monomer in solution (Fig. 5a and b). However, four LbcA(ΔN48) molecules formed an interlocked tetramer as a dimer of dimers in the crystal (Fig. 5c and d). The chamber of the TPR spiral of the first LbcA is occupied by the H1-H4 of a second LbcA molecule on the top and by the H2-H3 of a third LbcA molecule on the side (Fig. 5d). Therefore, there is a four-helix bundle inside the first LbcA TPR spiral. We suggest that this bundle may mimic a substrate and that the LbcA spiral may wrap around a substrate to target it to CtpA for degradation (Fig. 5e). In this scenario, the conserved and hydrophobic residues of the TPR-A helices lining the inner surface of the TPR spiral may participate in binding that substrate.

### The LbcA H1 is essential for binding to and activating the CtpA protease.

**FIG 6:**
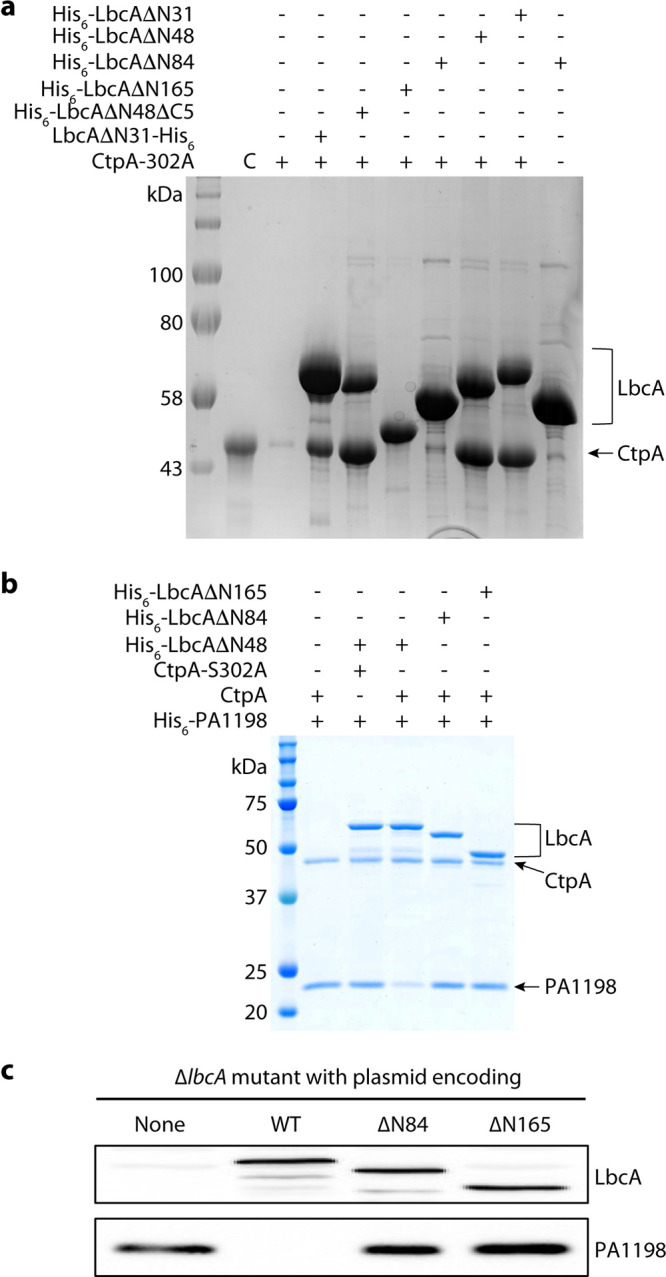
The LbcA N-terminal extension is essential for the protease activity of CtpA. (a) CtpA pulldown assay using various N-terminal and C-terminal deletion mutants of LbcA. Lane 2 is the CtpA input control (C). Lane 3 is the background binding of CtpA to nickel beads. (b) *In vitro* substrate (PA1198) degradation assay. (c) Substrate degradation *in vivo*. Plasmids encoding wild-type LbcA (WT), LbcA(ΔN84), or LbcA(ΔN165) were transformed into a P. aeruginosa ΔlbcA mutant. None = empty plasmid vector control. The LbcA proteins and accumulation of the PA1198 substrate were detected by immunoblot analysis with polyclonal antisera.

To identify the binding interface between CtpA and LbcA, we produced a series of N- and/or C-terminally truncated LbcA mutants and did a CtpA pulldown assay (Fig. 6a). We found that removing the C-terminal five residues of LbcA did not affect the LbcA-CtpA interaction. All constructs containing the complete H1 to H29 region pulled down CtpA. However, LbcA with either H1 truncation (His_6_-LbcAΔN84) or H1 to H4 truncation (His_6_-LbcAΔN165) failed to pull down CtpA. This result suggests that the LbcA N-terminal (NT) helical extension, in particular H1, is required for LbcA binding to CtpA, and that LbcA H1 directly participates in the binding.

We then carried out both *in vitro* and *in vivo* assays to monitor degradation of the PA1198 substrate (23). We first incubated purified CtpA with separately purified LbcA proteins and PA1198. As expected, PA1198 was degraded in the presence of His_6_-LbcAΔN48, which had the intact H1 (Fig. 6b). However, PA1198 was not degraded in the presence of His_6_-LbcA(ΔN84) (missing H1) or His_6_-LbcA(ΔN165) (missing H1 to H4). In fact, the outcomes of reactions with these two truncations were indistinguishable from those containing no LbcA or those using the inactive CtpA(S302) (Fig. 6b). We also constructed plasmids encoding LbcA with in-frame deletions equivalent to the ΔN84 or ΔN165 constructs but intact signal sequences, lipidation sites, and sorting signals at their N termini. In a Δ*lbcA* P. aeruginosa strain, these plasmids failed to activate CtpA as revealed by the accumulation of PA1198, which was present at a similar level as in a strain without any LbcA (Fig. 6c). Therefore, pulldowns and *in vitro* and *in vivo* activity assays pinpointed the LbcA H1 as a key binding element for activation of the CtpA protease.

## DISCUSSION

The LbcA–CtpA system supports the function of the P. aeruginosa type 3 secretion system, is required for virulence in a mouse model of acute infection, and affects surface attachment (10, 23). Furthermore, four CtpA substrates are PG cross-link hydrolases, which means that the LbcA-CtpA system affects the integrity of a crucial cell envelope component and perhaps the most important antibiotic target, the cell wall. Therefore, this system could be an effective antibiotic target, and the structural analysis reported here may aid the development of such antibiotics. Here, we have shown that CtpA assembles as a hexamer. However, the hexamer alone is inactive, because the catalytic triad Ser-302–Lys-327–Gln-331 is not in hydrogen-bonding distance. We also found that the CtpA partner protein LbcA has an N-terminal helical region that is needed to bind to CtpA and a large spiral cavity that has the capacity to wrap around a substrate for delivery to CtpA.

How the interaction with LbcA converts CtpA into an active protease is currently unclear. Elucidation of the activation mechanism requires the determination of the CtpA-LbcA complex. However, our previous experiments suggested that most, if not all, CtpA in the cell is bound to LbcA (23). CtpA fractionates with the membrane fraction in *lbcA*^+^ cells but is in the soluble periplasmic fraction in Δ*lbcA* cells, and when CtpA or LbcA is purified from P. aeruginosa, they copurify with a lot of the other one (23). Also, the pulldown assays done here show that LbcA and CtpA can interact in the absence of substrate (Fig. 6a). Therefore, it is possible that the LbcA-CtpA complex fluctuates between inactive and active states, and perhaps the presence of a substrate would stabilize the enzyme-adaptor substrate in the active state for productive substrate degradation.

The PDZ domain is the C-terminal peptide substrate-binding element of CTPs (32). The ability of PDZ domains to move in order to accommodate the incoming C-terminal substrate peptide partially accounts for the substrate delivery-based activation mechanism of the CTPs. The PDZ domain is the most flexible region in the CtpB structure and acts as an inhibitor by blocking the substrate peptide binding in the inactive form but moves away to form a narrow tunnel for a substrate peptide in the active form (18). Similarly, the PDZ domain of CtpA is highly flexible and largely invisible in the crystal structure of the inactive CtpA hexamer, suggesting a CtpA activation mechanism similar to that of CtpB. In the case of Prc from E. coli, the PDZ domain acts as an activator rather than an inhibitor, but its movement is still a key feature of the activation mechanism (8).

P. aeruginosa CtpA requires the partner lipoprotein LbcA for activation and targeted proteolysis (23). This is a variation in the general substrate activation mechanism. In this regard, the LbcA-CtpA system is analogous to the E. coli NlpI-Prc system, in which the TPR-containing adaptor NlpI plays the dual function of delivering the PG hydrolase MepS to the Prc protease for degradation and activating the protease (8, 15). Despite this functional similarity, significant differences exist between these two systems. The two proteases are in different C-terminal processing peptidase families; E. coli Prc is much larger than P. aeruginosa CtpA; and Prc functions as a monomer, not a hexamer. Therefore, the Prc N-terminal and C-terminal helical domains are not involved in oligomerization; instead, they wrap around the protease core, perhaps to limit substrate access to the protease. Nlpl has a total of 14 helices with four TPRs, compared with the 29 helices and 11 TPRs of LbcA. Although both NlpI and LbcA contain TPRs, the primary sequences of the proteins are not similar. Finally, NIpI functions as a dimer, unlike the monomeric state of LbcA.

The LbcA-CtpA system contributes to the virulence of P. aeruginosa. This makes understanding the structure and function of this proteolytic system broadly significant. Future challenges include understanding exactly how LbcA activates CtpA and how protein substrates, not just peptides, are specifically recognized by the multitude of CTPs and/or their adaptor proteins for tightly regulated proteolysis.

## MATERIALS AND METHODS

### Purification of CtpA.

DNA encoding amino acid 38 to the C terminus of CtpA was subcloned into plasmid pET15b between the NdeI and XhoI sites to encode N-terminal His_6_-tagged CtpA(ΔN37). Similar plasmids encoding CtpA-S302A, CtpA(ΔC6), CtpA-L426K L430K, or CtpA-L426A L430A were generated by site-directed mutagenesis. For all CtpA proteins, E. coli BL21(DE3) transformants were grown at 37°C to optical density at 600 nm (OD_600_) = 0.6 to 0.7 before being induced with 0.5 mM isopropyl-β-d-thiogalactopyranoside (IPTG) at 16°C overnight. Cells were lysed by passing through a microfluidizer cell disruptor in 10 mM potassium phosphate, pH 8.0, 10 mM imidazole, 0.25 M NaCl. The homogenate was clarified by centrifuging at 27,000 × *g*, and the supernatant was applied to a HiTrap-Ni column (GE Healthcare) preequilibrated with lysis buffer. Proteins were eluted with a 10 to 300 mM imidazole gradient in 10 mM potassium phosphate, pH 8.0, containing 0.25 M NaCl. Fractions containing His_6_-CtpA were collected. The N-terminal His tag was removed using thrombin (0.5 units/mg) by dialyzing against 20 mM Tris, pH 8.0, 150 mM NaCl overnight at 4°C. Untagged CtpA was further purified with HiTrap-Q in 10 mM Tris, pH 8.0, and a 50 to 500 mM NaCl gradient and polished by gel filtration in 10 mM Tris, pH 8.0, and 150 mM NaCl using Superdex 200 prep-grade column (16 × 600 mm, GE Healthcare).

### Purification of LbcA.

DNA encoding amino acid 49 to the C terminus of LbcA (LbcA[ΔN48]) was subcloned into the NdeI and HindIII sites of plasmid pET24b. Plasmids encoding various N-terminal His_6_-tagged LbcA proteins were constructed similarly by subcloning fragments into the NdeI and BamHI sites of plasmid pET15b. For all LbcA proteins, E. coli BL21(DE3) transformants were grown at 37°C to OD_600_ = 0.5 before being induced with 0.5 mM IPTG at 37°C for 3 h. His_6_-tagged LbcA protein was purified with HiTrap-Ni in 10 mM potassium phosphate, pH 8.0, 0.25 M NaCl, and a 10 to 300 mM imidazole gradient, followed by HiTrap-Q in 10 mM Tris, pH 8.0, and a 50 to 500 mM NaCl gradient. The final polish of LbcA was done in a Superdex 200 prep-grade column preequilibrated with 10 mM Tris, pH 8.0, and 150 mM NaCl.

### CtpA pulldown by LbcA.

Cell lysates of E. coli BL21(DE3) producing pET15b-encoded His_6_-tagged LbcA proteins were incubated with nickel-nitrilotriacetic acid-agarose (Ni-NTA-agarose) beads for 30 min at 4°C in 10 mM potassium phosphate, pH 8.0, 0.25 M NaCl, 10 mM imidazole. After washing with the same binding buffer, the His_6_-tagged LbcA proteins were incubated with 300 μg of untagged CtpA for 30 min at room temperature in 10 mM Tris, pH 8.0, 0.15 M NaCl, 10 mM imidazole. After washing with the same buffer, the proteins remaining on the Ni-NTA-agarose beads were analyzed by SDS-PAGE.

### Protein crystallization and structural solution.

All protein concentrations were determined by Bradford assay. CtpA(ΔN37) was crystallized at 20°C by the sitting-drop vapor-diffusion method using 0.1 M sodium acetate, pH 4.0, and 0.6 M ammonium dihydrogen phosphate at a concentration of 33 mg/mL. Diffraction data were collected at the Advanced Photon Source and processed with Mosflm software. Se-derived crystals diffracted to 3.3 Å and were used to solve the phases. Autosol in Phenix was used to locate Se sites, and the resulting map was used to build the initial model. Only four high-occupancy Se sites were identified in one asymmetric unit by SAD method. We determined the CtpA(ΔN37) structure by combining molecular replacement (the core domain of B. subtilis CtpB; PDB ID 4C2C) with SAD. The PDZ domain had weak density. Because CtpA and CtpB PDZs are homologous with 38% identity, we generated a CtpA PDB model based on CtpB PDZ structure, and rigid-body-fitted the model into the density map. We found that M153 and M160 coincide with the two low-occupancy Se sites, indicating the quality of the PDZ model. We next used the two Se sites as a guide to fine tune the PDZ rigid-body docking. Crystallization conditions for CtpA(ΔN37, S302A) were the same as wild-type CtpA, and the structure was solved using CtpA(ΔN37) as the search model.

LbcA(ΔN48) at a concentration of 45 mg/mL was crystallized at 20°C by the sitting-drop vapor-diffusion method using 0.1 M sodium acetate, pH 4.6, and 1.9 M ammonium dihydrogen phosphate. The diffraction data were collected at the Advanced Photon Source (APS) and were processed with Mosflm. The best data set was from Se-derived crystals which diffracted to 3.5 Å, so these data were used to solve the LbcA structure. Se sites were determined by SAD method using Autosol of Phenix (33). Atomic models were built in Coot (34) and refined by Phenix.refine (35). The crystal structures of CtpA, CtpA(S302A), and LbcA were deposited in the Protein Data Bank under accession codes 7RPQ, 7RQH, and 7RQF, respectively.

### *In vitro* proteolysis assay.

CtpA and LbcA were purified as described above. To purify His_6_-PA1198, E. coli strain M15 (pREP4) (Qiagen) containing pAJD2948 was grown in 500 mL of LB broth at 37°C to an OD_600_ of 0.6 to 1.0. Protein production was induced with 1 mM IPTG at 37°C for 3 h. His_6_-PA1198 was purified natively by Ni-NTA-agarose affinity chromatography in 50 mM NaH_2_PO_4_ and 300 mM NaCl, as recommended by the manufacturer (Qiagen). Protein was eluted in 50 mM NaH_2_PO_4_, 300 mM NaCl, 50 mM imidazole, pH 8. Assays were done as described previously (23, 36). The samples were separated by SDS-PAGE and stained with ProtoBlue Safe (National Diagnostics). For mutant CtpA protein experiments, ImageJ analysis was used to determine the densities of the His_6_-PA1198 bands. His_6_-PA1198 degradation was quantified by comparing His_6_-PA1198 band density to that in the reaction with inactive CtpA-S302A. Averages from two independent experiments are reported.

### *In vivo* CtpA activity assay.

Fragments encoding wild-type CtpA or CtpA-S302A were amplified from P. aeruginosa PAK or AJDP1140 DNA, respectively, using a primer annealing 39 bp upstream of the start codon and a reverse primer annealing immediately downstream of the stop codon. A fragment encoding CtpA(ΔC6) was generated with a reverse primer that annealed downstream of codon 430 and incorporated a stop codon. Fragments encoding CtpA-L426K L430K or CtpA-L426A L430A were generated with reverse primers incorporating the mutagenic mismatches. All fragments were cloned into pHERD26T using EcoRI and XbaI restriction sites added by the primers.

Fragments encoding LbcA(ΔN84) and LbcA(ΔN165) were generated by amplifying one fragment with a forward primer that annealed 38 bp upstream of the start codon and a reverse primer annealing at codon 31, and a second fragment with a forward primer annealing at codon 85 or codon 166 and a reverse primer annealing at the *lbcA* stop codon. The forward primer for the second fragments had a 5′ tail complementary to the end of the first fragment (codons 25 to 31). The first and second fragments were joined by splicing overlap extension PCR (37). The resulting fragments encoded LbcA with internal deletions that removed codons 32 to 84 (ΔN84) or codons 32 to 165 (ΔN165). Codons 1 to 31 were retained, encoding the signal sequence followed by 15 amino acids corresponding to the N terminus of mature wild-type LbcA, to facilitate normal signal sequence processing, lipidation, and outer membrane trafficking. A fragment encoding wild-type *lbcA* was generated by amplifying *lbcA* from P. aeruginosa DNA using a forward primer annealing 38 bp upstream of the start codon and a reverse primer annealing at the *lbcA* stop codon. All fragments were cloned into pHERD26T using EcoRI and HindIII restriction sites added by the primers.

Plasmids were introduced into Δ*ctpA* or Δ*lbcA* mutants by electroporation (38). Saturated cultures were diluted into 5 mL of LB broth containing 75 μg/mL tetracycline, in 18-mm diameter test tubes, at OD_600_ of 0.05. Cultures were grown on a roller drum at 37°C for 5 h. Cells harvested by centrifugation were resuspended in SDS-PAGE sample buffer at equal concentrations based on culture OD_600_. Samples were separated by SDS-PAGE and transferred to nitrocellulose by semidry electroblotting. Chemiluminescent detection followed incubation with polyclonal antisera against CtpA, LbcA, or PA1198, and then goat anti-rabbit IgG (Sigma) horseradish peroxidase conjugate.

## References

[1] Vollmer W, Blanot D, de Pedro MA. 2008. Peptidoglycan structure and architecture. FEMS Microbiol Rev 32:149–167. doi:10.1111/j.1574-6976.2007.00094.x. 18194336

[2] Pazos M, Peters K, Vollmer W. 2017. Robust peptidoglycan growth by dynamic and variable multi-protein complexes. Curr Opin Microbiol 36:55–61. doi:10.1016/j.mib.2017.01.006. 28214390

[3] Vollmer W. 2012. Bacterial growth does require peptidoglycan hydrolases. Mol Microbiol 86:1031–1035. doi:10.1111/mmi.12059. 23066944

[4] Park SH, Kim YJ, Lee HB, Seok YJ, Lee CR. 2020. Genetic evidence for distinct functions of peptidoglycan endopeptidases in *Escherichia coli*. Front Microbiol 11:565767. doi:10.3389/fmicb.2020.565767. 33013796 PMC7516022

[5] Singh SK, Parveen S, SaiSree L, Reddy M. 2015. Regulated proteolysis of a cross-link-specific peptidoglycan hydrolase contributes to bacterial morphogenesis. Proc Natl Acad Sci USA 112:10956–10961. doi:10.1073/pnas.1507760112. 26283368 PMC4568209

[6] Rawlings ND, Barrett AJ, Bateman A. 2010. MEROPS: the peptidase database. Nucleic Acids Res 38:D227–D233. doi:10.1093/nar/gkp971. 19892822 PMC2808883

[7] Ye F, Zhang M. 2013. Structures and target recognition modes of PDZ domains: recurring themes and emerging pictures. Biochem J 455:1–14. doi:10.1042/BJ20130783. 24028161

[8] Chueh CK, Som N, Ke LC, Ho MR, Reddy M, Chang CI. 2019. Structural basis for the differential regulatory roles of the PDZ domain in C-terminal processing proteases. mBio 10:e01129-19. doi:10.1128/mBio.01129-19. 31387902 PMC6686036

[9] Bandara AB, Sriranganathan N, Schurig GG, Boyle SM. 2005. Carboxyl-terminal protease regulates *Brucella suis* morphology in culture and persistence in macrophages and mice. J Bacteriol 187:5767–5775. doi:10.1128/JB.187.16.5767-5775.2005. 16077124 PMC1196076

[10] Seo J, Darwin AJ. 2013. The *Pseudomonas aeruginosa* periplasmic protease CtpA can affect systems that impact its ability to mount both acute and chronic infections. Infect Immun 81:4561–4570. doi:10.1128/IAI.01035-13. 24082078 PMC3837984

[11] Deng CY, Deng AH, Sun ST, Wang L, Wu J, Wu Y, Chen XY, Fang RX, Wen TY, Qian W. 2014. The periplasmic PDZ domain-containing protein Prc modulates full virulence, envelops stress responses, and directly interacts with dipeptidyl peptidase of *Xanthomonas oryzae* pv. *oryzae*. Mol Plant Microbe Interact 27:101–112. doi:10.1094/MPMI-08-13-0234-R. 24200074

[12] Carroll RK, Rivera FE, Cavaco CK, Johnson GM, Martin D, Shaw LN. 2014. The lone S41 family C-terminal processing protease in *Staphylococcus aureus* is localized to the cell wall and contributes to virulence. Microbiology 160:1737–1748. doi:10.1099/mic.0.079798-0. 24928312 PMC4117222

[13] Bandara AB, DeShazer D, Inzana TJ, Sriranganathan N, Schurig GG, Boyle SM. 2008. A disruption of *ctpA* encoding carboxy-terminal protease attenuates *Burkholderia mallei* and induces partial protection in CD1 mice. Microb Pathog 45:207–216. doi:10.1016/j.micpath.2008.05.005. 18614331

[14] Nash ZM, Cotter PA. 2019. Regulated, sequential processing by multiple proteases is required for proper maturation and release of *Bordetella* filamentous hemagglutinin. Mol Microbiol 112:820–836. doi:10.1111/mmi.14318. 31152610

[15] Su MY, Som N, Wu CY, Su SC, Kuo YT, Ke LC, Ho MR, Tzeng SR, Teng CH, Mengin-Lecreulx D, Reddy M, Chang CI. 2017. Structural basis of adaptor-mediated protein degradation by the tail-specific PDZ-protease Prc. Nat Commun 8:1516. doi:10.1038/s41467-017-01697-9. 29138488 PMC5686067

[16] Campo N, Rudner DZ. 2006. A branched pathway governing the activation of a developmental transcription factor by regulated intramembrane proteolysis. Mol Cell 23:25–35. doi:10.1016/j.molcel.2006.05.019. 16818230

[17] Rudner DZ, Losick R. 2002. A sporulation membrane protein tethers the pro-sigmaK processing enzyme to its inhibitor and dictates its subcellular localization. Genes Dev 16:1007–1018. doi:10.1101/gad.977702. 11959848 PMC152351

[18] Mastny M, Heuck A, Kurzbauer R, Heiduk A, Boisguerin P, Volkmer R, Ehrmann M, Rodrigues CD, Rudner DZ, Clausen T. 2013. CtpB assembles a gated protease tunnel regulating cell-cell signaling during spore formation in *Bacillus subtilis*. Cell 155:647–658. doi:10.1016/j.cell.2013.09.050. 24243021 PMC3808539

[19] Hoge R, Laschinski M, Jaeger KE, Wilhelm S, Rosenau F. 2011. The subcellular localization of a C-terminal processing protease in *Pseudomonas aeruginosa*. FEMS Microbiol Lett 316:23–30. doi:10.1111/j.1574-6968.2010.02181.x. 21204920

[20] Paterson DL. 2006. The epidemiological profile of infections with multidrug-resistant *Pseudomonas aeruginosa* and *Acinetobacter species*. Clin Infect Dis 43:S43–S48. doi:10.1086/504476. 16894514

[21] Tumbarello M, De Pascale G, Trecarichi EM, Spanu T, Antonicelli F, Maviglia R, Pennisi MA, Bello G, Antonelli M. 2013. Clinical outcomes of *Pseudomonas aeruginosa* pneumonia in intensive care unit patients. Intensive Care Med 39:682–692. doi:10.1007/s00134-013-2828-9. 23370828

[22] Crivaro V, Di Popolo A, Caprio A, Lambiase A, Di Resta M, Borriello T, Scarcella A, Triassi M, Zarrilli R. 2009. *Pseudomonas aeruginosa* in a neonatal intensive care unit: molecular epidemiology and infection control measures. BMC Infect Dis 9:70. doi:10.1186/1471-2334-9-70. 19463153 PMC2692859

[23] Srivastava D, Seo J, Rimal B, Kim SJ, Zhen S, Darwin AJ. 2018. A proteolytic complex targets multiple cell wall hydrolases in *Pseudomonas aeruginosa*. mBio 9:e00972-18. doi:10.1128/mBio.00972-18. 30018106 PMC6050968

[24] Graham JB, Canniff NP, Hebert DN. 2019. TPR-containing proteins control protein organization and homeostasis for the endoplasmic reticulum. Crit Rev Biochem Mol Biol 54:103–118. doi:10.1080/10409238.2019.1590305. 31023093 PMC6816462

[25] Zeytuni N, Zarivach R. 2012. Structural and functional discussion of the tetra-trico-peptide repeat, a protein interaction module. Structure 20:397–405. doi:10.1016/j.str.2012.01.006. 22404999

[26] Blatch GL, Lassle M. 1999. The tetratricopeptide repeat: a structural motif mediating protein-protein interactions. Bioessays 21:932–939. doi:10.1002/(SICI)1521-1878(199911)21:11<932::AID-BIES5>3.0.CO;2-N. 10517866

[27] Chakraborty D, Darwin AJ. 2021. Direct and indirect interactions promote complexes of the lipoprotein LbcA, the CtpA protease and its substrates, and other cell wall proteins in *Pseudomonas aeruginosa*. J Bacteriol 203:e0039321. https://doi/org/10.1128/JB.00393-21. doi:10.1128/JB.00393-21. 34570626 PMC8604077

[28] Ntountoumi C, Vlastaridis P, Mossialos D, Stathopoulos C, Iliopoulos I, Promponas V, Oliver SG, Amoutzias GD. 2019. Low complexity regions in the proteins of prokaryotes perform important functional roles and are highly conserved. Nucleic Acids Res 47:9998–10009. doi:10.1093/nar/gkz730. 31504783 PMC6821194

[29] Konovalova A, Silhavy TJ. 2015. Outer membrane lipoprotein biogenesis: Lol is not the end. Philos Trans R Soc B 370:20150030. doi:10.1098/rstb.2015.0030. PMC4632606 26370942

[30] Lewenza S, Mhlanga MM, Pugsley AP. 2008. Novel inner membrane retention signals in *Pseudomonas aeruginosa* lipoproteins. J Bacteriol 190:6119–6125. doi:10.1128/JB.00603-08. 18641140 PMC2546801

[31] Narita S, Tokuda H. 2007. Amino acids at positions 3 and 4 determine the membrane specificity of *Pseudomonas aeruginosa* lipoproteins. J Biol Chem 282:13372–13378. doi:10.1074/jbc.M611839200. 17350956

[32] Clausen T, Kaiser M, Huber R, Ehrmann M. 2011. HTRA proteases: regulated proteolysis in protein quality control. Nat Rev Mol Cell Biol 12:152–162. doi:10.1038/nrm3065. 21326199

[33] Adams PD, Afonine PV, Bunkoczi G, Chen VB, Echols N, Headd JJ, Hung LW, Jain S, Kapral GJ, Grosse Kunstleve RW, McCoy AJ, Moriarty NW, Oeffner RD, Read RJ, Richardson DC, Richardson JS, Terwilliger TC, Zwart PH. 2011. The Phenix software for automated determination of macromolecular structures. Methods 55:94–106. doi:10.1016/j.ymeth.2011.07.005. 21821126 PMC3193589

[34] Casanal A, Lohkamp B, Emsley P. 2020. Current developments in Coot for macromolecular model building of electron cryo-microscopy and crystallographic data. Protein Sci 29:1069–1078. doi:10.1002/pro.3791. 31730249 PMC7096722

[35] van Zundert GCP, Moriarty NW, Sobolev OV, Adams PD, Borrelli KW. 2021. Macromolecular refinement of X-ray and cryoelectron microscopy structures with Phenix/OPLS3e for improved structure and ligand quality. Structure 29:913–921.e4. doi:10.1016/j.str.2021.03.011. 33823127 PMC8349848

[36] Chung S, Darwin AJ. 2020. The C-terminus of substrates is critical but not sufficient for their degradation by the *Pseudomonas aeruginosa* CtpA protease. J Bacteriol 202:e00174-20. doi:10.1128/JB.00174-20. PMC8404705 32482720

[37] Heckman KL, Pease LR. 2007. Gene splicing and mutagenesis by PCR-driven overlap extension. Nat Protoc 2:924–932. doi:10.1038/nprot.2007.132. 17446874

[38] Choi KH, Kumar A, Schweizer HP. 2006. A 10-min method for preparation of highly electrocompetent *Pseudomonas aeruginosa* cells: application for DNA fragment transfer between chromosomes and plasmid transformation. J Microbiol Methods 64:391–397. doi:10.1016/j.mimet.2005.06.001. 15987659

