# Supplementary material for: Pseudomonas aeruginosa C-Terminal Processing Protease CtpA Assembles into a Hexameric Structure That Requires Activation by a Spiral-Shaped Lipoprotein-Binding Partner
Source: mBio. 2022 Jan 18;13(1):e03680-21. doi: 10.1128/mbio.03680-21 (PMC8764530; doi:10.1128/mbio.03680-21)
Supplement: TABLE S1 [file mbio.03680-21-st001.pdf]

Nam	Genotype/Features	Reference or Source
***P. aeruginosa* strains**		
PAK	wild-type PAK strain	(1)
AJDP730	PAK Δ*ctpA*	(2)
AJDP1091	PAK Δ*lbcA::aacC1*	(2)
AJDP1140	PAK *ctpA-S302A*	(2)
***E. coli* strain**		
BL21(DE3)	F-$^-$ *ompT gal [dcm] [lon]* $hsdS_B$ (r_B^- m_B^-; *E. coli* B strain) λDE3	(3)
**Plasmids**		
pET15b	Amp^r, pMB1 *ori*, *T7p* expression vector	Novagen
pET24b	Kan^r, pMB1 *ori*, *T7p* expression vector	Novagen
pHERD26T	Tet^r, pMB1 *ori*, *araBp* expression vector	(4)
pQE-30	Amp^r, Col E1 *ori*, *T5p* expression vector	Qiagen
pAJD2948	*T5p-his$_6$*-PA1198 in pQE-30	(5)
pAJD3037	*araBp-ctpA* in pHERD26T	This study
pAJD3038	*araBp-ctpAΔC6* in pHERD26T	This study
pAJD3039	*araBp-ctpA-L426K L430K* in pHERD26T	This study
pAJD3045	*araBp-ctpA-S302A* in pHERD26T	This study
pAJD3063	*araBp-ctpA-L426A L430A* in pHERD26T	This study
pAJD3109	*araBp-lbcA* in pHERD26T	This study
pAJD3110	*araBp-lbcAΔN84* in pHERD26T	This study
pAJD3111	*araBp-lbcAΔN165* in pHERD26T	This study
pET15b_CtpAΔN37	*T7p-his$_6$-ctpAΔN37* in pET15b	This study
pET15b_CtpAΔN37ΔC6	*T7p-his$_6$-ctpAΔN37ΔC6* in pET15b	This study
pET15b_CtpAΔN37_S302A	*T7p-his$_6$-ctpAΔN37_S302A* in pET15b	This study

pET15b_CtpAΔN37_L426K/L430K	*T7p-his*$_6$*-ctpAΔN37_L426K L430K* in pET15b	This study
pET15b_CtpAΔN37_L426A/L430A	*T7p-his*$_6$*-ctpAΔN37_L426A L430A* in pET15b	This study
pET24b_LbcAΔN48	*T7p-lbcAΔN48-his*$_6$ in pET24b	This study
pET15b_LbcAΔN31	*T7p-his*$_6$*-lbcAΔN31* in pET15b	This study
pET15b_LbcAΔN48	*T7p-his*$_6$*-lbcAΔN48* in pET15b	This study
pET15b_LbcAΔN84	*T7p-his*$_6$*-lbcAΔN84* in pET15b	This study
pET15b_LbcAΔN165	*T7p-his*$_6$*-lbcAΔN165* in pET15b	This study
pET15b_LbcAΔN48ΔC5	*T7p-his*$_6$*-lbcAΔN48ΔC5* in pET15b	This study

REFERENCES

1. Strom MS, Lory S. 1986. Cloning and expression of the pilin gene of *Pseudomonas aeruginosa* PAK in *Escherichia coli*. J Bacteriol 165:367-372.
2. Srivastava D, Seo J, Rimal B, Kim SJ, Zhen S, Darwin AJ. 2018. A Proteolytic Complex Targets Multiple Cell Wall Hydrolases in *Pseudomonas aeruginosa*. mBio 9.
3. Studier FW, Rosenberg AH, Dunn JJ, Dubendorff JW. 1990. Use of T7 RNA polymerase to direct expression of cloned genes. Methods in Enzymology 185:60-89.
4. Qiu D, Damron FH, Mima T, Schweizer HP, Yu HD. 2008. PBAD-based shuttle vectors for functional analysis of toxic and highly regulated genes in *Pseudomonas* and *Burkholderia* spp. and other bacteria. Appl Environ Microbiol 74:7422-7426.
5. Chakraborty D, Darwin AJ. 2021. Direct and indirect interactions promote complexes of the lipoprotein LbcA, the CtpA protease and its substrates, and other cell wall proteins in *Pseudomonas aeruginosa*. J Bacteriol doi:10.1128/JB.00393-21:JB0039321.
