# Supplementary material for: Pseudomonas aeruginosa C-Terminal Processing Protease CtpA Assembles into a Hexameric Structure That Requires Activation by a Spiral-Shaped Lipoprotein-Binding Partner
Source: mBio. 2022 Jan 18;13(1):e03680-21. doi: 10.1128/mbio.03680-21 (PMC8764530; doi:10.1128/mbio.03680-21)
Supplement: TABLE S2 [file mbio.03680-21-st002.pdf]

	CtpAΔN37_Se	CtpA(ΔN37, S302A)	LbcAΔN48_Se
**Data collection**			
Wavelength (Å)	0.97931	1.07803	0.97872
Space group	R 3	R 3	P 32 2 1
Cell dimensions			
a, *b*, *c* (Å)	187.49, 187.49, 132.01	189.65, 189.65, 131.10	120.70, 120.70, 221.94
α, β, γ (°)	90.00, 90.00, 120.00	90.00, 90.00, 120.00	90.00, 90.00, 120.00
Resolution (Å)	93.75-3.3(3.48-3.3)*	94.82-3.2(3.37-3.2)*	94.57-3.5(3.69-3.5)*
R_{merge} (%)	16.7 (91.2)	8.9 (142)	18.3 (226)
$I / \sigma I$	10.8 (2.9)	15.6 (2.0)	13.2 (2.0)
Total reflections	301,572 (44,008)	297,808 (44,906)	536,619 (78,420)
Completeness (%)	100 (100)	99.6 (99.9)	100 (100)
Redundancy	11.6 (11.4)	10.3 (10.6)	22.1 (22.5)
Wilson B-factor	91.13	113.98	137.72
**Refinement**			
Resolution (Å)	39.83 – 3.30	43.03 – 3.2	60.39 – 3.50
No. reflections	26,026	28,849	24,277
R_{work} / R_{free}	0.2375 / 0. 2636	0.2422/0.2654	0.2470 / 0.2806
No. of non-hydrogen atoms	3933	3931	8352
Macromolecule	3933	3931	8352
Ligand	0	0	0
Water	0	0	0
B-factors	91.58	126.34	135.73
Macromolecule	91.58	126.34	135.73
Ligand			
Water			
R.m.s. deviations			
Bond lengths (Å)	0.003	0.003	0.003
Bond angles (°)	0.757	0.739	0.608
Ramachandran statistics (%)			
Favored	96.83	97.02	97.89
Allowed	3.17	2.98	2.11
Outliers	0	0	0

*Values in parentheses are for the last (highest) resolution shell.
