# Supplementary figures and images for: Pseudomonas aeruginosa C-Terminal Processing Protease CtpA Assembles into a Hexameric Structure That Requires Activation by a Spiral-Shaped Lipoprotein-Binding Partner
Source: mBio. 2022 Jan 18;13(1):e03680-21. doi: 10.1128/mbio.03680-21 (PMC8764530; doi:10.1128/mbio.03680-21)

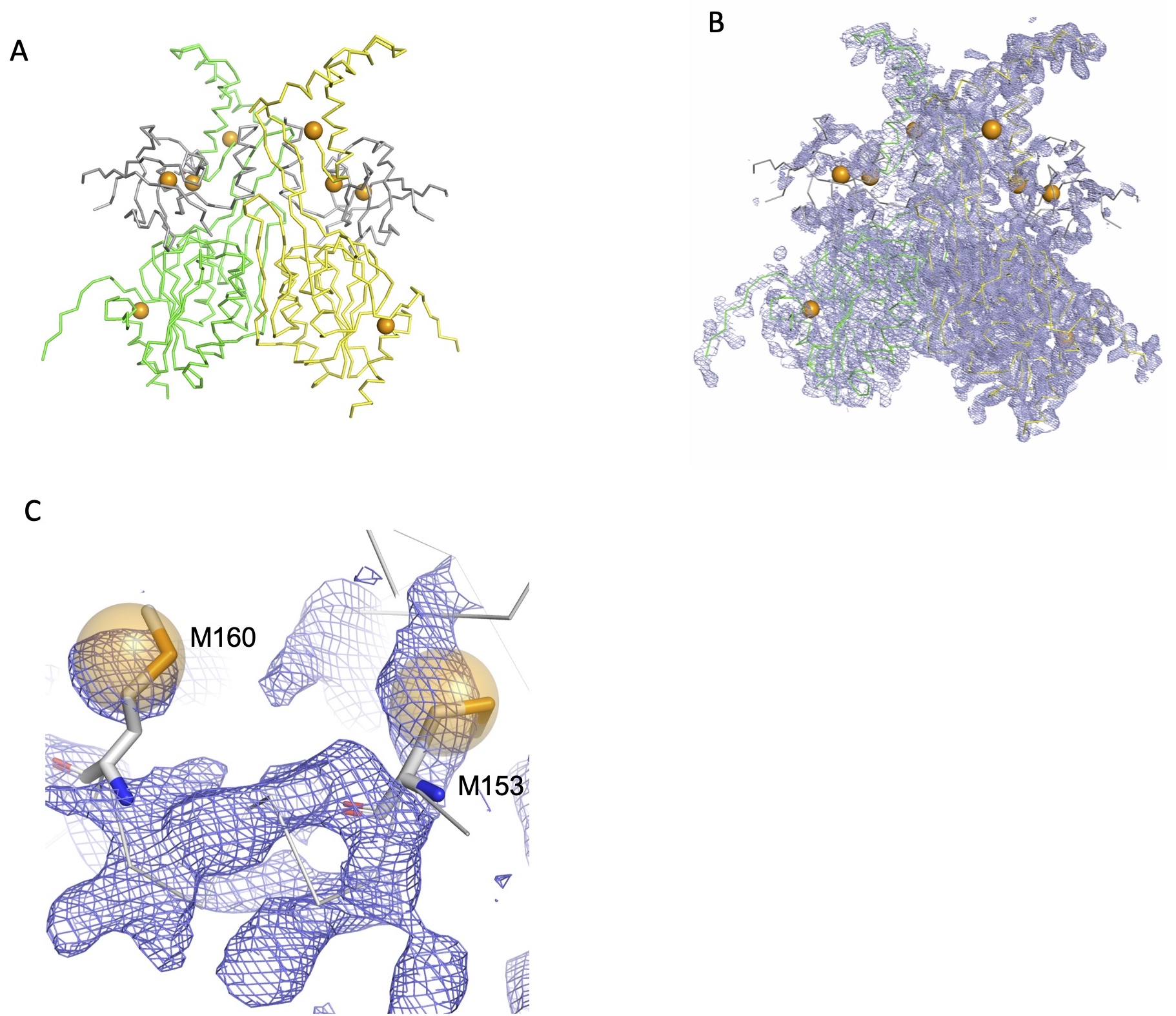

Supplement: FIG S1 [file mbio.03680-21-sf001.jpg]

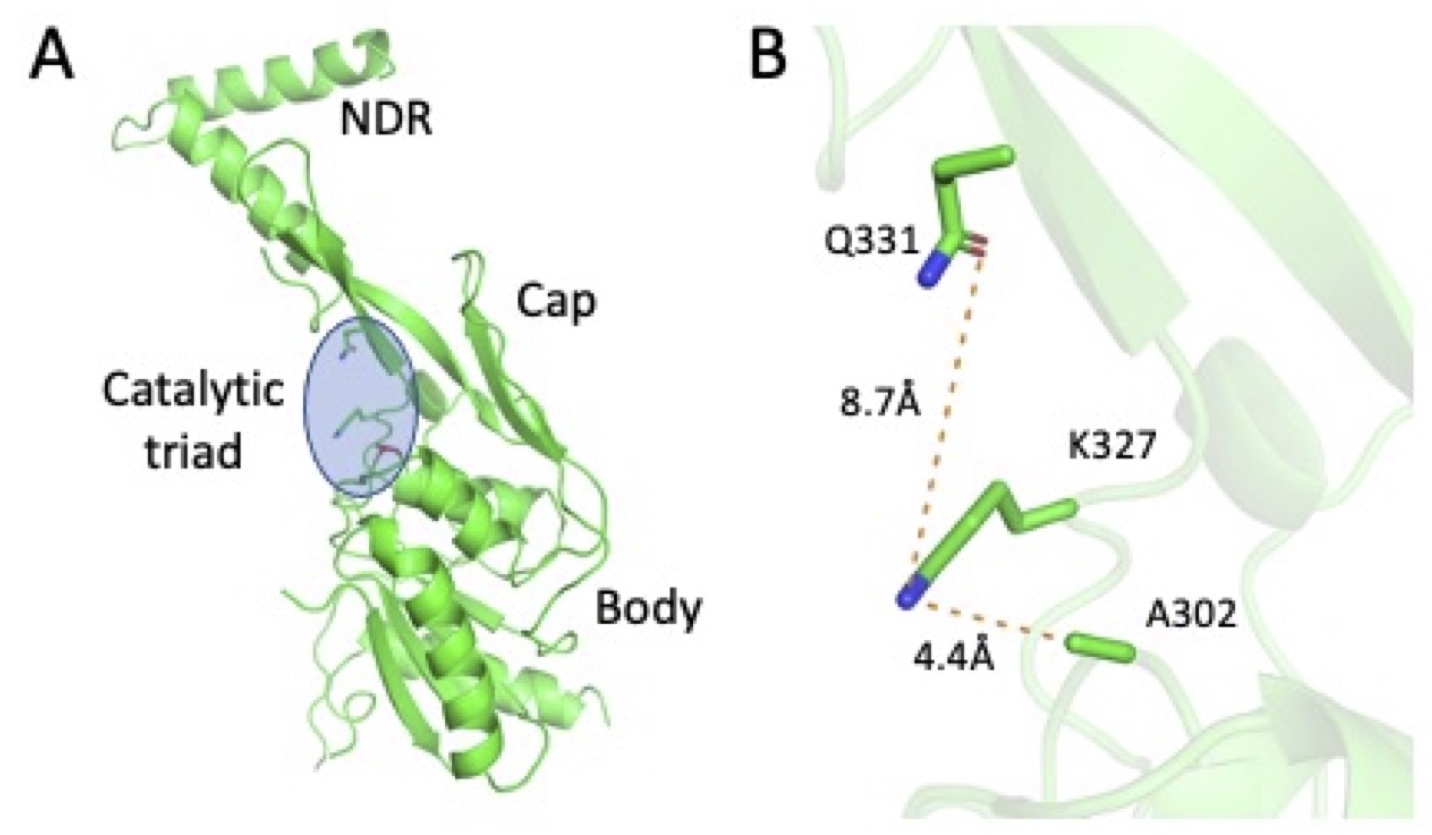

Supplement: FIG S2 [file mbio.03680-21-sf002.jpg]
